# Population structure and genetic diversity of the endangered fish black shinner *Pseudopungtungia nigra* (Cyprinidae) in Korea: a wild and restoration population

**DOI:** 10.1038/s41598-023-36569-4

**Published:** 2023-06-15

**Authors:** Kang-Rae Kim, Yeong-Ho Kwak, Mu-Sung Sung, Seong-Jang Cho, In-Chul Bang

**Affiliations:** 1 Department of Life Science & Biotechnology, Soonchunhyang University, Asan, 31538 Republic of Korea; 2 Boryeong Freshwater Eco Center, Boryeong, 33416 Korea

**Keywords:** Genetics, Molecular biology

## Abstract

The black shinner *Pseudopungtungia nigra* Mori, 1935 is an endangered fish endemic to Korea. It lives in the narrow basin of the Geumgang River, Mangyeonggang River, and Ungcheoncheon Stream, which flow into the West Sea of Korea. One population of *P. nigra* in Ungcheoncheon Stream has been locally exterminated once; it is now inhabiting the upper reaches of the dam through a restoration program. Efforts to identify and understand the genetic structure of these populations are important for conservation planning. Here, we analyzed genetic diversity using 21 microsatellite markers for 9 populations. The mean number of alleles ranged from 4.4 to 8.1, mean allelic richness ranged from 4.6 to 7.8, mean observed heterozygosity ranged from 0.519 to 0.702, and mean expected heterozygosity ranged from 0.540 to 0.763. All groups had recent and historical bottlenecks (*P* < 0.05, M-ratio < 0.68). Three groups [YD (2019), OC and UC] had significant inbreeding index values, suggesting that they were engaged in inbreeding. We observed a moderate level of genetic differentiation between MG and the rest of the population (*F*_ST_ = 0.135 to 0.168, *P* < 0.05). The genetic structure exhibited a fitting constant *K* = 2, along with separation between MG and the remaining populations. With respect to genetic flow, YD (2019), OC, CG, and ND shifted to the UC population (0.263 to 0.278). The genetic flow of each population was transferred only within the population; there was no gene flow among populations, except for the Ungcheoncheon Stream population. This study shows that the Ungcheoncheon Stream population needs conservation efforts to increase its genetic diversity, and the Geumgang River populations needs a conservation plan that considers the possibility of conservation and evolution through gene exchange among the populations.

## Introduction

Biodiversity has exhibited greater decline in freshwater ecosystems than in terrestrial and marine ecosystems^1–3^. Because of human activities and the expansion of living areas toward rivers, numerous freshwater fish have become endangered via habitat fragmentation (e.g. by dams and weirs), pollution, and overfishing^4–6^. In particular, fish living in rivers are sensitive to the effects of physical, chemical, and biological factors on aquatic environments,rapid changes to environmental conditions increase the possibility of species extinction^7–9^. Therefore, restoration plans have been established to prevent the extinction of endangered species^10–13^.

In the past, genetic management was not considered when designing restoration plans^12^. However, the need for genetic management has been continuously advocated for the efficient restoration of endangered species^14,15^. Accordingly, genetic technologies have been used and improved in conservation planning in recent decades, but the application of genetic management remains insufficient because of awareness and cost issues^13,16,17^.

Microsatellites have specific sequence repeats, are widely distributed throughout the genome, and have high polymorphism; thus, they are widely used in conservation studies of endangered species to investigate genetic diversity, genetic structure, bottlenecks, and genetic flow among populations^18–26^.

The black shinner *Pseudopungtungia nigra* Mori, 1935 is an endangered species endemic to Korea, which belongs to the Cypriniformes order, Cyprinidae family, and Gobioninae subfamily. It is found only on the Korean Peninsula and is narrowly distributed in the water systems of the Geumgang River, Ungcheoncheon Stream, and Mangyeonggang River^27,28^. The black shinner is highly regarded as an ornamental fish because of its streamlined body and black spot pattern on the tail, it is at risk of overfishing because of human activities. Considering its narrow distribution and risk of overfishing, the black shinner has been designated as first-class endangered wildlife in Korea^29^. The fish mainly lives in rocky or stony places with good water quality,it breeds by brood parasitism in the spawning grounds of *Coreoperca herzi* and is thus found together with *C. herzi*^27^.

In the Ungcheoncheon Stream water system, a *P. nigra* habitat was identified prior to the construction of the Boryeong dam (1992), but it was not found anywhere upstream or downstream after the construction of the dam; thus, it is considered extinct in this region (Ministry of Environment^30^). In contrast, the construction of the Daecheong and Yongdam dams in the Geumgang River water system, where *P. nigra* is widely distributed, has led to habitat fragmentation; the construction of continuous weirs continues to cause increasing fragmentation. Accordingly, a release project for restoration was conducted to prevent the extinction of fish in the upper stream of Gapcheon, a tributary of the Geumgang River water system, and in the Ungcheoncheon Stream downstream of the Boryeong dam (Ministry of Environment^30^). However, the population was not restored because of failed habitat adaptation, except in a narrow portion of the Ungcheoncheon Stream in the upper reaches of the Boryeong dam. The Ungcheoncheon Stream population is presumed to have used the Geumgang River water system population for restoration, but this speculation cannot be confirmed because there is no official record. Reintroductions are important tools for the recovery of endangered species^31^. Effective reintroduction programs in endangered fish populations require extensive knowledge of their genetic structure^32^. Genetic diversity and bottlenecks in reintroduced populations must be carefully considered for species restoration^14,33,34^. Although restoration was successful, factors such as genetic diversity were not considered in the restoration program at the time. Generally, conservation programs for endangered species have many issues to consider related to genetic factors, such as inbreeding in declining populations^35,36^. In the absence of prior knowledge regarding genetic background, perturbations of the genetic structure of the restoration population may have adverse effects^37,38^. The identification of genetic diversity and genetic structure in the Ungcheoncheon Stream population restored by reintroduction can provide important insights for conservation planning. Thus, there is a need to clarify the origin during efforts to conserve the genetic diversity of the Ungcheoncheon Stream population.

Genetic diversity is the driving force that allows species to adapt to their environment and maintain their evolutionary potential^14,39,40^. Habitat fragmentation limits gene flow, thereby increasing genetic inbreeding^14,41^. Increased inbreeding causes inbreeding depression, and positive feedback from inbreeding depression increasingly accelerates population extinction^42–44^. Currently, the population of *P. nigra* is in a state of habitat fragmentation related to the construction of dams and weirs; it has already been exterminated once in the Ungcheoncheon Stream water system. Because the restored Ungcheoncheon population is isolated, it is likely to experience inbreeding depression in the absence of outside intervention. Genetic studies of some populations in the Geumgang and Mangyeonggang Rivers were conducted using amplified fragment length polymorphism markers to establish a restoration plan^45^. However, there is a lack of information regarding genetic diversity and structure of the overall population of *P. nigra*; such information is necessary for comprehensive conservation management plans. Moreover, efficient conservation planning requires genetic studies of restoration and isolated populations.

In the present study, genetic diversity was analyzed for the Geumgang River water system population, Ungcheoncheon Stream population (restored population), and Mangyeonggang River population; the Yudeungcheon Stream (2012) and Mangyeonggang River (2008) populations were also subjected to genetic monitoring analyses. Additionally, the genetic structure was evaluated to determine the distributions of genetic differentiation and variation at the species and population levels; the genetic flow was analyzed to provide information regarding the origin of the restored Ungcheoncheon population (UC). Overall genetic information regarding this species will provide the basis for conservation and management plans for endangered *P. nigra*.

## Materials and methods

### Sampling and DNA extraction

**Figure 1:**
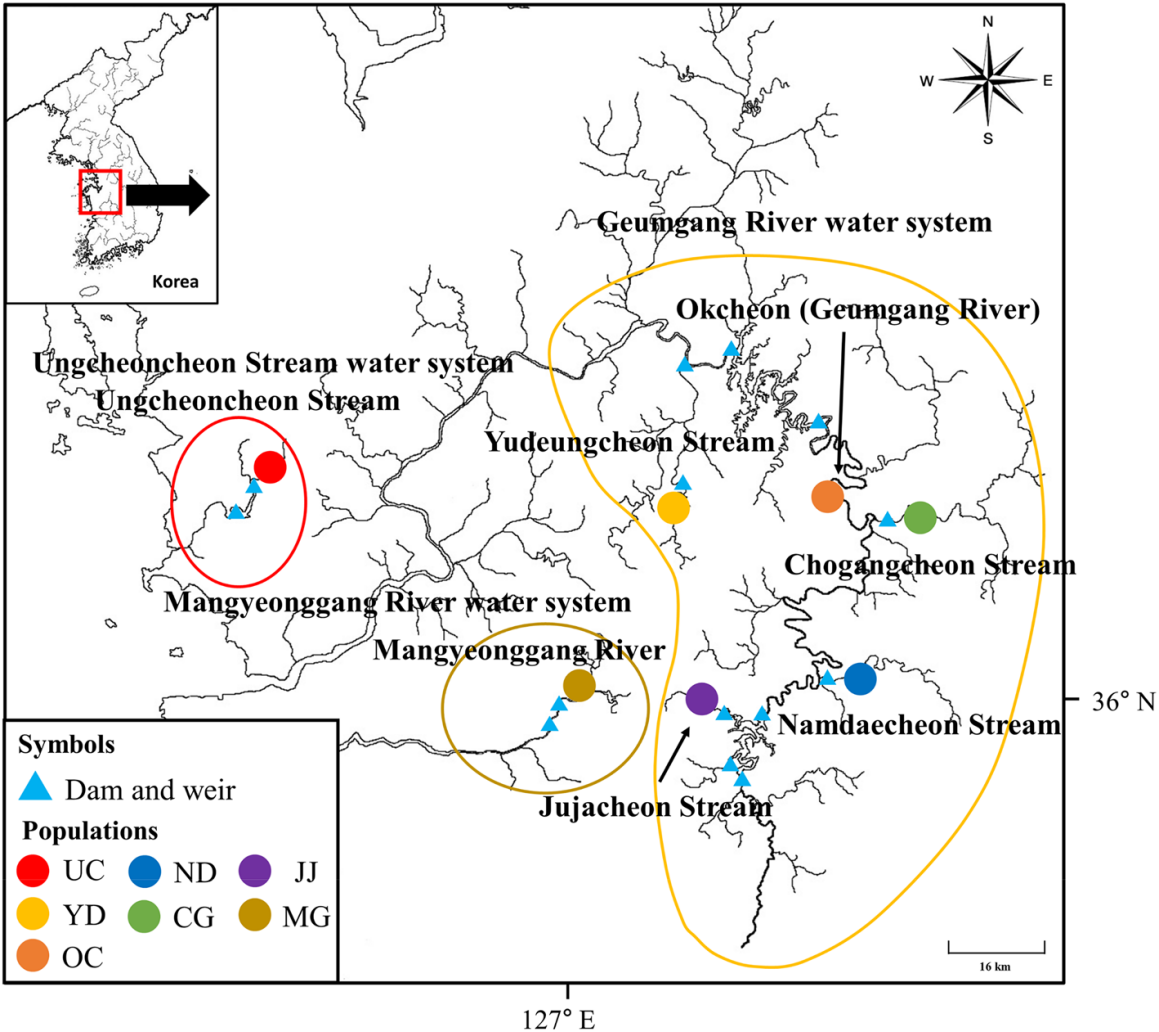
Locations of seven sites where black shiner fish were collected for genetic analysis. Location information for dams, and weirs. Circled areas indicate different water systems.

*P. nigra* Mori, 1935 is an endangered species in Korea and was collected with permission from the Geum River Basin Environmental Office (permit nos.: 2018-35, 2019-26) and Jeonbuk Regional Environmental Office of the Ministry of Environment (permit nos.: 2018-16, 2019-15). The sampled areas were collected as shown in Fig. 1 between March and November 2019 (Geumgang River, Ungcheoncheon Stream, and Mangyeonggang River water systems). For the *P. nigra* samples, we used pectoral fin tissue to obtain at least 30 DNA samples (Supplementary Table S1). DNA samples previously collected from the Yudengcheon (2012) and Mankyunggang (2008) populations were included in the genetic diversity analysis because they are isolated populations that require genetic monitoring. The remaining populations were excluded from genetic monitoring analysis because they did not have DNA samples available. Genomic DNA was extracted using the Genomic DNA Prep Kit (BioFact, Seoul, South Korea), in accordance with the manufacturer’s protocol. Tissue samples were stored in 99% ethanol until DNA extraction. DNA samples were diluted to 50 ng/µL with deionized water and stored at − 80 °C.

### Multiplex PCR and genotyping

The microsatellite markers developed in a previous study^46^ constituted four multiplex PCR sets for efficient genotyping and reduced analysis costs (Supplementary Table S2). Multiplex PCR reactions were performed in sets of four; forward primers were labeled with 6-carboxyfluorescein (FAM), hexachlorofluorescein (HEX), and tetramethylrhodamine (TAMRA). The PCR reaction comprised a total volume of 20 µL, including 50 ng of genomic DNA and forward and reverse primers at concentrations of 0.1 µM, along with the Multiplex PCR Premix (Bioneer Inc., Daejeon, South Korea). The following thermocycler conditions were used: initial denaturation at 94 °C for 5 min; 34 cycles of denaturation at 94 °C for 30 s, annealing at 57.5 °C for 30 s, and extension at 72 °C for 30 s; and final elongation at 72 °C for 7 min. Amplified products were identified by electrophoresis on a 1.5% agarose gel. For genotyping, 1 µL of tenfold diluted PCR product was mixed with Hi-Di formamide (Thermo Fisher Scientific, Waltham, MA, USA) and 500 LIZ size standard (Applied Biosystems, Waltham, MA, USA), denatured at 95 °C for 5 min, then analyzed on an ABI 3730xl Analyzer (Applied Biosystems). The size of the allele was determined by scoring for each marker using Peak Scanner software (Ver. 1.0; Applied Biosystems); MICRO-CHECKER software (Ver. 2.2.3)^47^ was used to evaluate the presence or absence of scoring errors in markers.

### Genetic diversity and bottleneck analyses

Genetic diversity was measured by number of alleles (*N*_A_), expected heterozygosity (*H*_E_), and observed heterozygosity (*H*_O_) using CERVUS software (Ver. 3.0)^48^. Allelic richness (*AR* was analyzed using FSTAT software (Ver. 2.9.3)^49^ to correct for population-level differences. Analyses of population inbreeding coefficient (*F*_IS_ and Hardy–Weinberg equilibrium (HWE deviations were performed using GENEPOP software (Ver. 4.2)^50^ and ARLEQUIN software (Ver. 3.5)^51^.

Two methods were used to estimate bottlenecks. The first method used BOTTLENECK software (Ver. 1.2.02)^52^, a program for estimating bottlenecks via heterozygous excess testing using the infinite allele model^53^, two-phase model, and stepwise mutation model^54^. Each model was run for 10,000 iterations, and significance was determined using the Wilcoxon signed-rank test^55^. The second method used the M-ratio^56^, which estimates bottlenecks by using the mean ratio of the range of allele numbers and allele sizes,this analysis was performed using ARLEQUIN (Ver. 3.5)^51^. To determine the size of the effective population, LDNE software^57^ was used for linkage disequilibrium estimation.

### Population genetic structure and gene flow analysis

Genetic distance was analyzed using Nei’s minimum distance method^58^ in the Genclass 2 software (Ver. 2.0)^59^. Genetic differentiation (F_ST_ analysis and analysis of molecular variance (AMOVA were conducted using ARLEQUIN (Ver. 3.5)^51^. For cluster analysis, principal coordinates analysis (PCoA) based on genetic distance was performed using GenAlEx software (Ver. 6.50)^60^.

Genetic structure analysis was performed with STRUCTURE software (Ver. 2.3)^61^ using a Bayesian method of model-based clustering. The assignment test was performed via clustering between individuals, the no-admixture method, an appropriate model, was applied to unmixed water systems. To determine the most appropriate population, the population constant (K) was determined in the range of 1–10. Clustering was performed repeatedly with a burn-in length of 50,000; Markov chain Monte Carlo analysis was repeated with 100,000 iterations. To estimate the population constant (K), cluster results corresponding to the appropriate K values were analyzed with STRUCTURE HARVESTER^62^. Discriminant analysis of principal components (DAPC) was conducted to analyze gene clusters using the Adegenet package in R (Ver. 2.1.3)^63^, on a non-model basis without prior information about populations.

Migration rates among populations were measured using BayesAss software (Ver. 3.0.4)^64^. The Markov chain Monte Carlo method was used for 10,000,000 iterations, with a burn-in length of 1,000,000.

The model that best explains the statistical origin of the present reintroduced population was identified using DIYABC software (Ver. 2.1.0)^65^. An approximate Bayesian computational approach (ABC) was used to evaluate the posterior probabilities of past scenarios. Five populations from the Geumgang River water system were combined and divided into three groups, the Mangyeonggang River population and the UC population, to evaluate origin scenarios for reintroduction (UC) history (Fig. 1).

### Ethical approval

All experimental protocols were approved by the designated Soonchunhyang University and the permission committee of the Ministry of Environment, Korea (permit nos.: 2018-35, 2019-26, 2018-16, 2019-15). We declare that all methods were performed in accordance with Soonchunhyang University and the Ministry of Environment guidelines and regulations. In addition, all experiments were performed in accordance with ARRIVE related guidelines and Soonchunhyang University regulations. Samples by population were collected according to the guidelines of Soonchunhyang University and the Ministry of Environment, Korea.

## Results

### Genetic diversity and bottlenecks

Among the developed microsatellite markers^46,66^, 25 microsatellite loci were successfully amplified and used to establish at least four markers per multiplex PCR set. The multiplex PCR assays consisted of seven-loci (Set1) and six-loci (Set2, Set3, and Set4) reaction systems. Information and product sizes for the four multiplex PCR sets are presented in Supplementary Table S1. Of the 25 microsatellites, 21 were included in the population analysis, and four with PIC < 0.3 were excluded.

Genetic diversity information for nine populations of *P. nigra* is shown in Table 1. With respect to the 21 microsatellite markers used for genetic diversity, no evidence of linkage disequilibrium was present after Bonferroni correction. The number of mean alleles per locus for the entire population ranged from 4.4 to 8.1 (Table 1). The mean allelic richness (*AR*) ranged from 4.6 to 7.8, mean observed heterozygosity (*H*_O_) ranged from 0.519 to 0.702, and mean expected heterozygosity (*H*_E_) ranged from 0.540 to 0.763; these results indicated moderate genetic diversity. Among the nine populations, the CG, OC, and ND populations had high genetic diversity compared with the other populations, whereas the MG (2019 and 2008) populations had low genetic diversity. The YD (2019) population had increased genetic diversity compared with the previously sampled YD (2012) population (*H*_O_ = 0.661 and 0.653, respectively); the MG (2019) population also had higher genetic diversity than the previously sampled MG (2008) population (*H*_O_ = 0.551 and 0.519, respectively). Notably, the UC population, a population restored after extinction in the Ungcheoncheon Stream, had moderate genetic diversity (*H*_O_ = 0.659).

**Table 1:** Population genetic diversity summary of *Pseudopungtungia nigra* based on 21 microsatellite loci.

Group	*N*	*N*_A_	*AR* (n = 19)	*H*_O_	*H*_E_	*P*_HWE_	*F*_IS_
OC	30	8.1	7.8	0.680	0.763	0.000***	0.102***
ND	30	7.8	7.2	0.669	0.736	0.000***	0.026
CG	30	7.0	6.9	0.702	0.724	0.000***	0.043
YD (2019)	30	6.8	6.5	0.661	0.732	0.000***	0.083***
YD (2012)	21	6.0	6.0	0.653	0.704	0.099	0.024
JJ	30	5.3	5.2	0.667	0.686	0.000***	0.023
UC	30	6.9	6.7	0.659	0.740	0.000***	0.096***
MG (2019)	30	4.5	4.8	0.551	0.540	0.040*	− 0.019
MG (2008)	19	4.4	4.6	0.519	0.582	0.568	0.025
All	250	6.3	6.2	0.640	0.689	0.000***	0.062

OC Okcheon stream in the Geumgang river water system, ND Namdaecheon stream in the Geumgang river water system, CG Chogangcheon stream in the Geumgang river water system, YD (2019) Yudeungcheon stream sampled from 2019 in the Geumgang river water system, YD (2012) Yudeungcheon stream sampled from 2012 in the Geumgang river water system, JJ Jujacheon stream in the Geumgang river water system, UC Ungcheoncheon stream water system, MG (2019) Mangyeonggang river water system sampled in 2019, MG (2008) Mangyeonggang river water system sampled in 2008, *N*: number of samples, *N*_A_ number of alleles, *AR* allelic richness, *H*_E_ expected heterozygosity, *H*_O_ observed heterozygosity, *P*_HWE_ extracted P-value estimated by Fisher’s exact test in the Markov chain Monte Carlo method, *F*_IS_ inbreeding coefficient, *P < 0.05, **P < 0.01, ***P < 0.001.

Analysis using Fisher’s exact test revealed that all populations except the YD (2012) and MG (2008) populations deviated from HWE (*P* < 0.05). The range of inbreeding coefficients was − 0.019 to 0.102 in the entire population, with high inbreeding signals for the OC (0.102), YD (0.083), and UC (0.096) populations (*P* < 0.001).

Recent bottleneck estimations showed that all populations experienced bottlenecks in the infinite allele model (*P* < 0.05). The MG (2019) and ND population bottlenecks were statistically significant (*P* < 0.05) in both the infinite allele model and stepwise mutation model. Mode-shift analysis revealed evidence of a bottleneck in the MG (2008) population.

The M-ratio, representing historical bottlenecks, ranged from 0.440 to 0.482 in all populations; Garza and Williamson^56^ suggested that values < 0.68 indicate evidence of a bottleneck. All populations experienced bottlenecks from previous generations to the present (M < 0.68). Effective population size ranged from 52 (MG 2008) to 301 (OC) (Table 2. The effective population size of the YD (2019 population was 190; the effective population size of YD (2012 could not be estimated. The effective population size of MG (2019 increased to 236 from 52 (MG 2008. However, these results are presumably errors related to the size of the sampled populations; *N*_e_ did not reach a size sufficient to maintain the minimum viable population (*N*_e_ < 1000).

**Table 2:** Summary statistics regarding bottleneck signatures, effective population size, and estimated M-ratios for nine *P. nigra* populations, *N* number of samples,* N*_e_ effective population size.

Population ID	*N*	Bottleneck tests				*N*_e_^^^	(95% CI)	M-ratio
		P_IAM_	P_TPM_	P_SMM_	Mode-shift			
OC	30	0.000***	0.330	0.327	L-SHAPE	301	(134–∞)	0.472
ND	30	0.001*	0.014*	0.001**	L-SHAPE	–	(319–∞)	0.467
CG	30	0.008**	0.485	0.350	L-SHAPE	74	(48–148)	0.440
YD (2019)	30	0.000***	0.186	0.096	L-SHAPE	190	(96–∞)	0.456
YD (2012)	21	0.007**	0.501	0.096	L-SHAPE	–	(150–∞)	0.471
JJ	30	0.001**	0.326	0.335	L-SHAPE	191	(103–1018)	0.442
UC	30	0.000***	0.489	0.330	L-SHAPE	74	(53–119)	0.482
MG (2019)	30	0.038*	0.227	0.018*	L-SHAPE	236	(90–∞)	0.449
MG (2008)	19	0.004**	0.495	0.224	SHIFTED	52	(31–137)	0.447

P_IAM_ P-value of bottleneck tests using infinite allele mutation model, P_TPM_ P-value of bottleneck tests using two-phase mutation model (10% variance and 90% proportion of SMM), P_SMM_ P-value of bottleneck tests using stepwise mutation model (SMM), Ne^^^ estimated effective population size according to LDNE software, *CI* confidence interval, OC Okcheon stream in the Geumgang river water system, ND Namdaecheon stream in the Geumgang river water system, CG Chogangcheon stream in the Geumgang river water system, YD (2019) Yudeungcheon stream sampled from 2019 in the Geumgang river water system, YD (2012) Yudeungcheon stream sampled from 2012 in the Geumgang river water system, JJ Jujacheon stream in the Geumgang river water system, UC Ungcheoncheon stream water system, MG (2019) Mangyeonggang river water system sampled in 2019, MG (2008) Mangyeonggang river water system sampled in 2008, *P < 0.05, **P < 0.01, ***P < 0.001.

### Population structure and estimation of gene flow

Table 3 shows the results of genetic distance and genetic differentiation analyses using microsatellite markers. For all populations, the between-population genetic distance ranged from 0.016 to 0.136; it was high between MG and the remaining seven populations (pairwise distance > 0.112, pairwise genetic differentiation > 0.135). Notably, the YD (2019) and JJ populations showed the greatest genetic distance and differentiation from the MG (2019) population (pairwise distance > 0.136, pairwise genetic differentiation > 0.161). However, the geographically isolated UC population of the Ungcheoncheon Stream water system showed very low genetic differentiation from populations of the Geumgang River water system, except for the MG (2019) population. Although there generally were large genetic differences between geographically isolated populations, the UC population appeared to be similar to the Geumgang River water system population because of low genetic distance and genetic divergence. This result suggests that the nine populations can be divided into two groups: Geumgang River and Ungcheoncheon Stream water system population vs. Mangyeonggang River water system population.

**Table 3:** Pairwise genetic distance and *F*_st_ among *P. nigra* populations according to microsatellite analysis.

Population	OC	ND	CG	YD (2019)	JJ	UC	MG (2019)
OC	–	0.016	0.025	0.037	0.040	0.016	0.115
ND	0.003	–	0.024	0.038	0.038	0.016	0.136
CG	0.016	0.015	–	0.031	0.050	0.024	0.112
YD (2019)	0.030	0.033	0.024	–	0.053	0.038	0.136
JJ	0.036	0.035	0.051	0.054	–	0.035	0.136
UC	0.003	0.003	0.014	0.032	0.030	–	0.114
MG (2019)	0.135	0.136	0.136	0.161	0.168	0.136	–

Pairwise distance (above),* F*_st_ pairwise genetic differentiation (below), OC Okcheon stream in the Geumgang river water system, ND Namdaecheon stream in the Geumgang river water system, CG Chogangcheon stream in the Geumgang river water system, YD Yudeungcheon stream in the Geumgang river water system, JJ Jujacheon stream in the Geumgang river water system, UC Ungcheoncheon stream water system, MG Mangyeonggang river water system.

**Figure 2:**
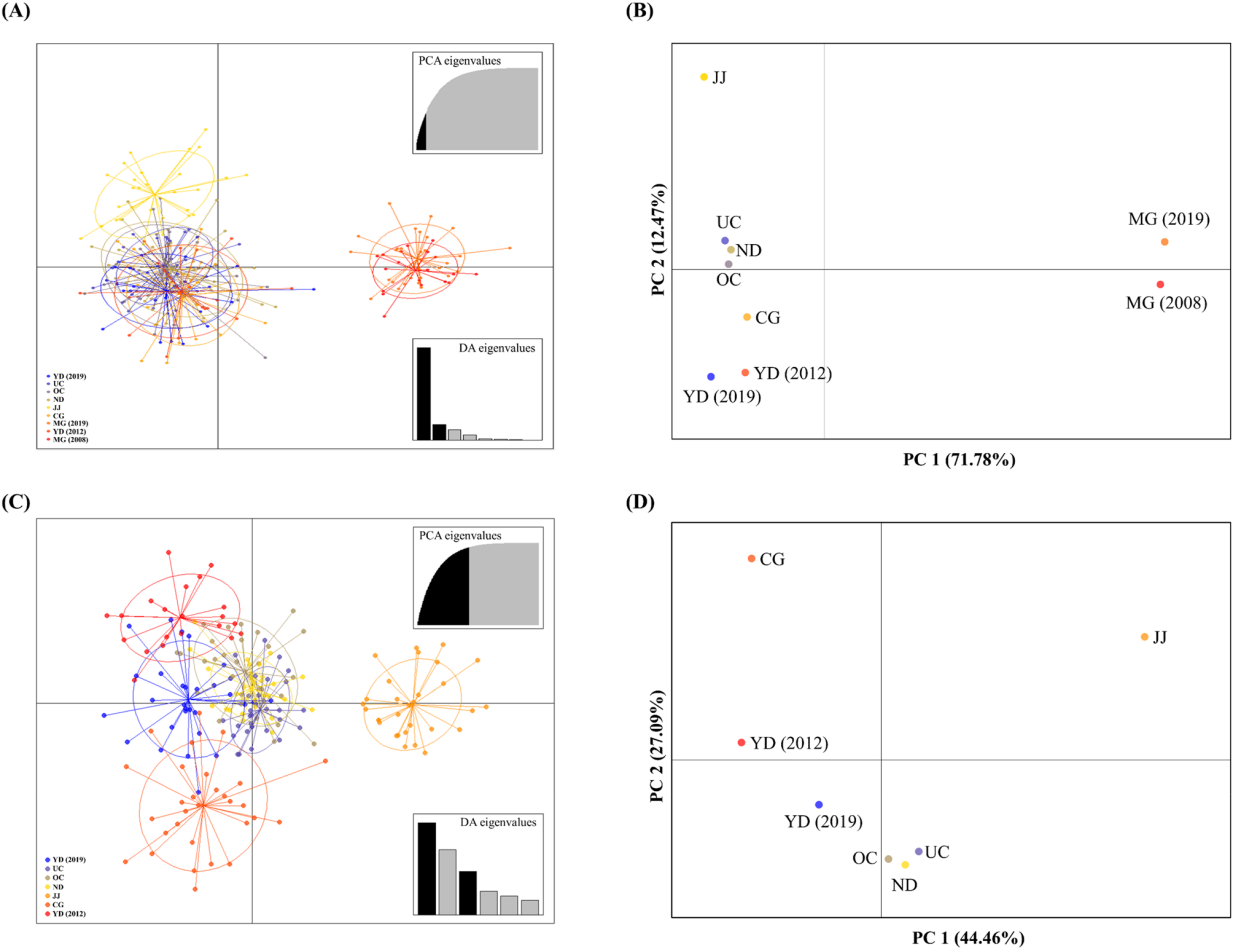
Scatterplots of discriminant analysis of principal components (DAPC) and principal coordinates analysis (PCoA) for 250 *Pseudopungtungia nigra* individuals. (**A**) DAPC plot for nine populations. (**B**) PCoA plot for nine populations. (**C**) DAPC plot for seven populations in the Geumgang river and Ungcheoncheon stream water system. (**D**) PCoA plot for seven populations in the Geumgang river and Ungcheoncheon stream water system.

PCoA and DAPC plots showed that the nine populations were divided into two groups, similar to the results of genetic differentiation (Fig. 2). The first group consisted of the populations of the Geumgang River and Ungcheoncheon Stream water systems, whereas the second group consisted of the MG (2019 and 2008) populations (Fig. 2). Except for MG (2019), the PCoA and DAPC results of the Geumgang River and Ungcheoncheon Stream water systems appeared to be divided into three groups. The first group comprised the OC, ND, JJ, CG, YD (2019), and YD (2012) populations; the second group comprised the JJ population; and the third group comprised the CG population.

**Figure 3:**
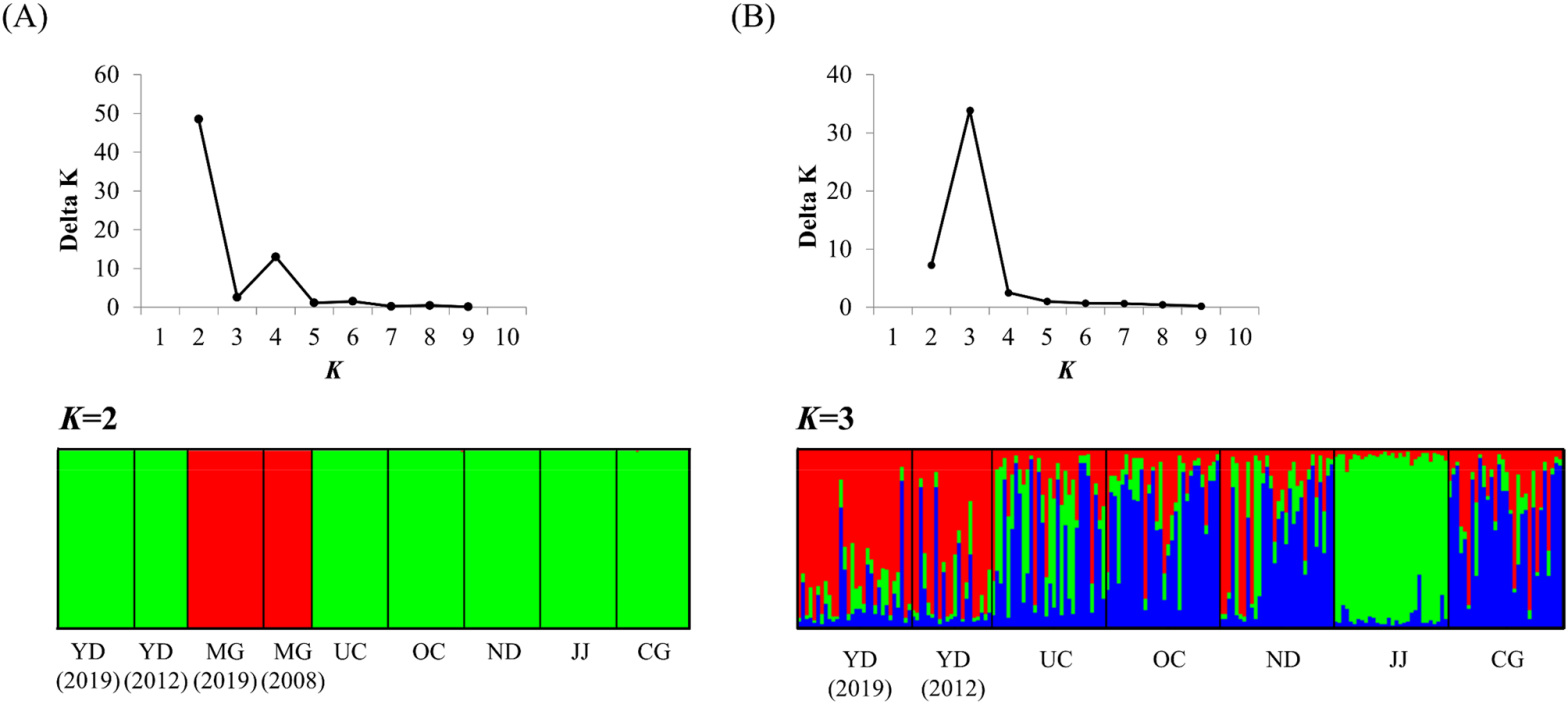
Population genetic structure of *P. nigra*. Each bar graph represents a population, and the color range of each bar represents its probability of assignment to a particular cluster. (**A**) Genetic structure for all populations. (**B**) Genetic structure for seven populations in the Geumgang river and Ungcheoncheon stream water systems.

The optimal number of populations (*K*) for all populations was determined to be *K* = 2, according to the Bayesian clustering method in STRUCTURE (Fig. 3). The populations were assigned to two groups: the Geumgang River and Ungcheoncheon Stream water systems [OC, ND, CG, YD (2019), JJ, and UC], and the remaining MG (2019 and 2008) populations. The STRUCTURE results for the Geumgang River and Ungcheoncheon Stream water system populations showed *K* = 3, although genotypes were mixed.

Based on these overall results (genetic distance, PCoA, DAPC, *F*_ST_, and STRUCTURE), the Geumgang River population [OC, ND, CG, YD (2019 and 2012), JJ], UC population, and Mangyeonggang River population [MG (2019 and 2008)] were separated and AMOVA was performed. AMOVA of the entire group divided the nine populations into two groups; the within-group variation was 86.23%, and the between-group variation was 11.26%. A significant genetic difference was present between the two groups at *F*_ST_ = 0.060 (*P* < 0.001).

When the Geumgang River and Ungcheoncheon Stream water systems were regarded as three groups, the within-group variation was 96.77%, and the between-group variation was 1.29% (Table 4). There was no significant between-group variance (*F*_CT_ = 0.013, *P* > 0.05). The genetic variation between the Geumgang River and UC populations was lower than that between the MG and Geumgang River and UC populations, suggesting that the genetic variation between the Geumgang and UC populations was not large.

**Table 4:** Summary statistics for analysis of molecular variance among and within populations of *P. nigra.*

Source of variation	d.f.	Sum of squares	Variance components	Percentage of total variance	*F*-statistics
Water system grouping (MG (2019, 2008) vs OC, ND, YD (2019, 2012), JJ, CG and UC)					
Among groups	1	170.971	0.974	11.57	*F*c_T_ = 0.189**
Among populations within groups	7	126.169	0.185	2.20	*F*_SC_ = 0.025***
Within populations	491	3561.132	7.261	86.23	*F*_ST=_0.060***
Total	499	3858.272	8.420	100.00	
Regional grouping (OC, ND, YD (2019, 2012) and UC vs JJ vs CG)					
Among groups	2	53.062	0.101	1.29	*F*c_T_ = 0.013
Among populations within groups	4	66.348	0.151	1.93	*F*_SC_ = 0.020***
Within populations	395	2987.381	7.572	96.77	*F*_ST=_0.070***
Total	401	3106.791	7.824	99.99	

*F*_ST_ is based on standard permutation across the full data set.

*d.f*. degrees of freedom, ***P < 0.001.

**Figure 4:**
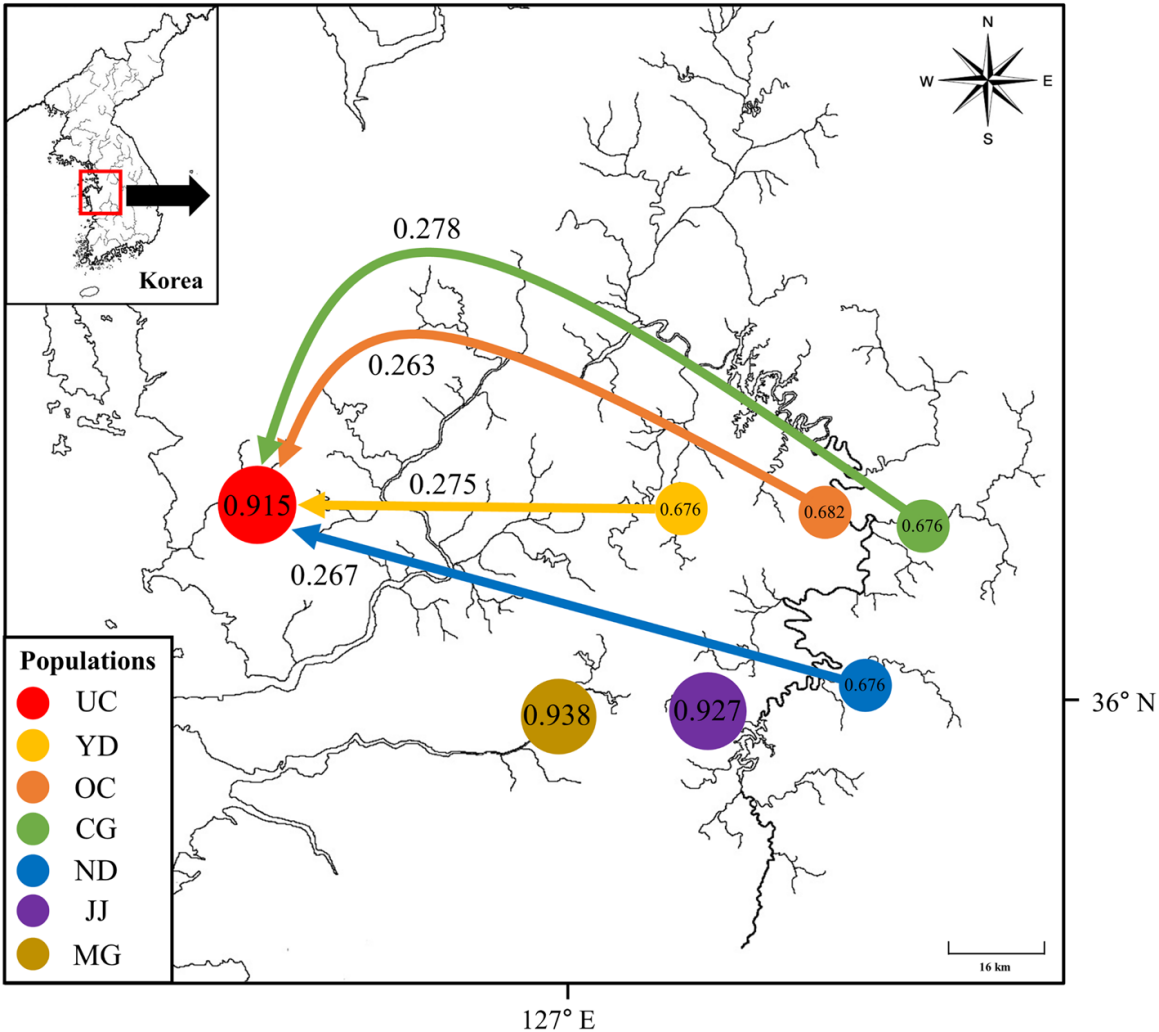
Genetic flow among and within *P. nigra* populations. Each circle represents a population; arrows and numbers indicate gene flow direction and gene flow rate.

Genetic flow was plotted using BayesAss software (Fig. 4). The highest migration rate was observed in the UC population; rates were 0.263 in the OC population, 0.267 in the ND population, 0.278 in the CG population, and 0.275 in the YD (2019) population. Our gene flow and dispersal results suggest that there is migration in the Geumgang River water system populations toward the UC population.

The genetic flow among populations, except for gene flow to the UC population, ranged from 0.003 to 0.031; these findings demonstrated that most gene flow occurred within each population, rather than among populations (Fig. 4). Overall, these results indicated isolation and the absence of genetic flow among populations, with the exception of the UC population.

**Figure 5:**
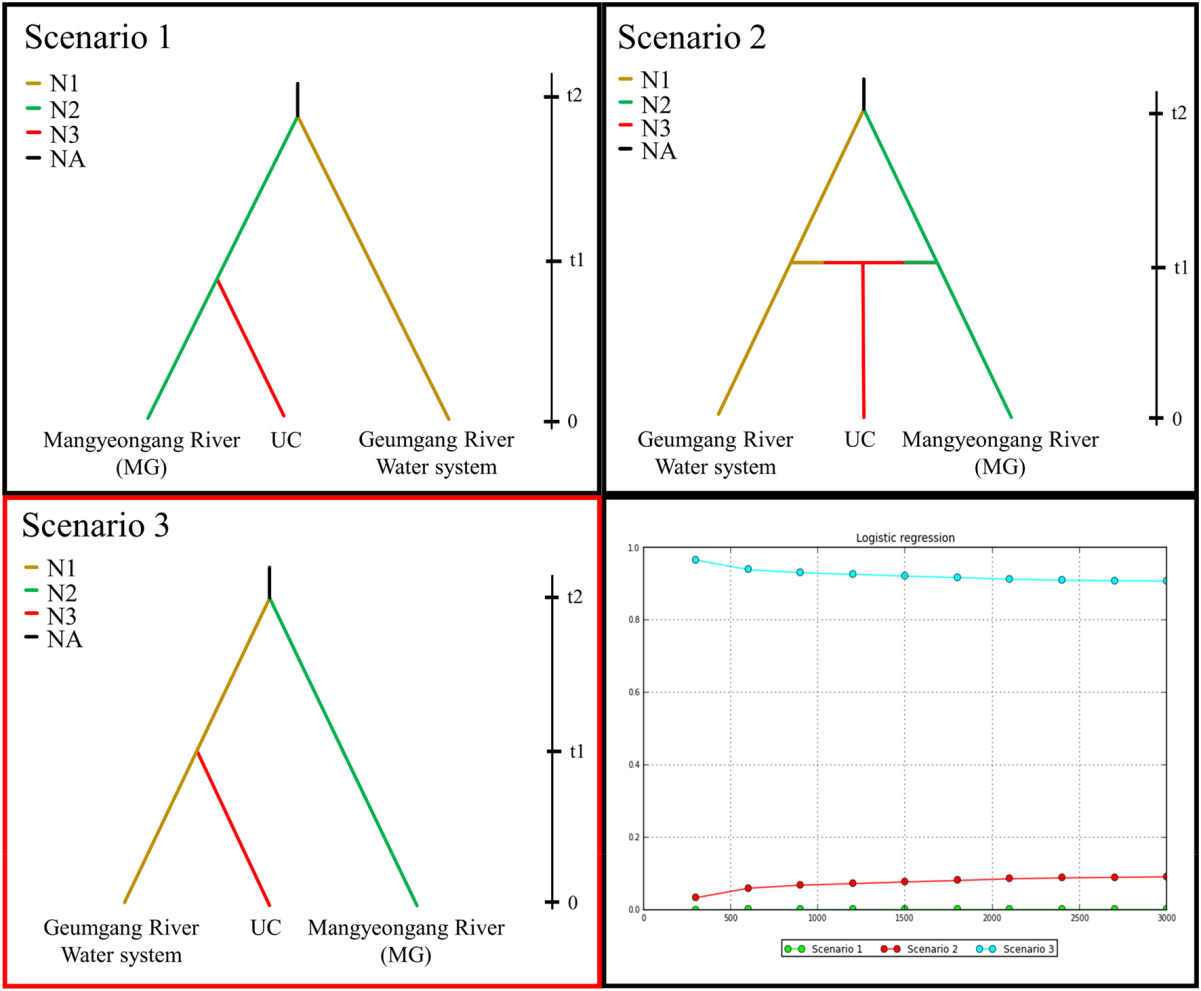
Assessment of population history scenarios for the UC population of *P. nigra* using approximate Bayesian calculation (ABC) inference. The best supported scenario is indicated with a red square.

ABC analysis was performed to determine the origin of the UC population of *P. nigra* (Fig. 5). Scenario 3, in which the UC population was derived from the Geumgang River population, showed the highest posterior probability of 0.9077 (Table 5). Scenarios in which the population was derived from the Mangyeonggang River (Scenario 1) or from both the Geumgang and Mangyeonggang Rivers (Scenario 2) had very low posterior probabilities (0.0009 and 0.0914).

**Table 5:** Comparison of three population history scenarios for *P. nigra* using DIYABC.

Scenario	Posterior probability (logistic regression)	95% confidence interval
1	0.0009	[0.0000, 0.0036]
2	0.0914	[0.0652, 0.1176]
3	**0.9077**	[0.8813, 0.9340]

Bold: high posterior probability.

## Discussion

In this study, *P. nigra*, an endemic and endangered fish in Korea, was subjected to analyses of genetic diversity and population structure in nine populations based on 21 microsatellite loci. The entire global population of the black shinner species inhabits only a small portion of the Korean peninsula; thus, conservation efforts and interests are high. Accordingly, this genetic study of endangered *P. nigra* provides essential genetic structure information for conservation.

### Genetic diversity and bottlenecks

Generally, endangered species tend to have low genetic diversity because of various factors such as genetic drift or inbreeding^14,67^. However, in the present study, despite its endangered species status, *P. nigra* had high genetic diversity similar to other freshwater fish. Kim et al.^45^ found similar results, with AFLP having higher genetic diversity than other endangered species (*lksookimia choii,* average genetic diversity: 0.086). Similar to the present study, high genetic diversity was observed in the freshwater and endangered fishes *Percocypris pingi*, yellow catfish (*Pelteobagrus fulvidraco*), roughskin sculpin (*Trachidermus fasciatus*), and golden mahseer (*Tor putitora*)^38,68–70^. Genetic diversity allows species to adapt and survive in changing environments by counteracting the effects of genetic drift^71^. Species with high genetic diversity are likely to maintain their high genetic diversity, even if they are endangered, unless the population size declines through events such as habitat destruction^68^. Therefore, it is presumed that high genetic diversity is maintained at the species level due to the wide distribution and high abundance of Geumgang River populations compared to the other populations.

We found high genetic diversity at the species level, but low genetic diversity in the MG (2019 and 2008) populations. The amplified fragment length polymorphism results of Kim et al.^45^ also supported these findings. Kim et al.^45^ found that MG populations have low genetic diversity and require genetic management. Because the MG population is geographically independent from the Geumgang River water system population and has a narrow geographical distribution compared with the Geumgang River water system population, it has a potential risk of genetic diversity loss. Therefore, for genetic management, the population should be augmented by breeding genetically identical MG populations.

In contrast, the UC population did not exhibit low genetic diversity similar to the Mangyeonggang River water system, although it is also a geographically independent population. *P. nigra* in the Ungcheoncheon Stream water system has been previously extinct and subsequently restored. There is no official record of the breeding population used to restore the Ungcheoncheon Stream water system, although the Geumgang River water system population was reportedly used. Therefore, the high genetic diversity of the UC population may be related to this aspect of restoration. A possible conservation strategy would be to augment populations with high genetic diversity. Although the UC population is genetically diverse because it lives in two small regions, the long-term effects of inbreeding cannot be ignored. Therefore, population augmentation is necessary to increase the genetic diversity of the population and reduce inbreeding.

The M-ratio and heterozygosity methods can detect genetic bottlenecks over long and short periods of time, respectively^56,72^. In the present study, all populations of *P. nigra* had recently experienced bottlenecks; the estimated M-ratio was below the critical value (M = 0.68), confirming signs of historical bottlenecks in all populations. The populations of *P. nigra* may have been affected by anthropogenic activities, such as the construction of dams and weirs in their habitat, which may have caused recent bottlenecks. However, the analysis of bottlenecks can produce biased results due to differences in population size, gene flow, number of samples, and number of loci. Thus, a larger number of samples should be analyzed using a more reliable assay. However, the indications of bottlenecks in the present study suggest that conservation efforts are needed in current populations.

Genetic monitoring of the YD and MG populations revealed increased genetic diversity over time. These results may have been biased because of the small number of samples. The consequences of these biases can be minimized with more sampling strategies and should be carefully considered for future results. Because the monitoring of populations with low genetic diversity is important for species conservation, continuous genetic monitoring must be conducted to conserve and enhance the gene pool.

Hardy–Weinberg equilibrium assumes that the number of frequencies remains constant from one generation to the next. This principle is based on five assumptions: no mutations occur, no migration occurs, the population is infinitely large, mating is random, and natural selection does not occur^73^.

Deviations from HWE can affect these assumptions and microsatellite alleles vary considerably in size, leading to scoring errors due to limitations of markers and sizing techniques^73^. OC, YD, and UC have a significant *F*_IS_, indicating that random breeding is unlikely to have occurred. Therefore, deviations from the HWE may occur. Inbreeding increases the probability of lethal genes because of genetic homozygosity and genetic diversity loss, hindering population maintenance^43,44^. Because genetic diversity is high, short-term effects may not be significant, however, continuous inbreeding causes inbreeding depression, resulting in the loss of genetic diversity and hindering active adaptation to environmental changes^14^. MG (2018) found that *F*_IS_ appears as a negative coefficient, indicating the possibility of outbreeding. This may have caused the deviation from the HWE. JJ ND, and CG could not find allelic stuttering, excessive heterozygosity, or homozygosity causing HWE deviation. A sufficient sample size is needed to prevent errors due to these HWE dropouts; therefore, it is necessary to obtain samples from different groups and ages at a single time point.

The M-ratio can detect genetic bottlenecks over a longer period of time, whereas heterozygosity methods can detect bottlenecks over shorter intervals^56,72^. In this study, all populations of *P. nigra* had recently experienced bottlenecks; the estimated M-ratio was also below the critical value (M = 0.68), confirming signs of historical bottlenecks in all populations. All populations of *P. nigra* may have been affected by anthropogenic activities such as the construction of dams and weirs in their habitat, which may have caused recent bottlenecks. However, the detection of bottlenecks can have biased results related to population size, gene flow, number of samples, and number of loci. Thus, there is a need to analyze a larger number of samples for a more reliable assay. However, indications of bottlenecks in this study suggest that conservation efforts are needed in current populations.

Effective population size (*N*_e_) randomly fluctuates according to population survival and reproduction, such that smaller population size increases the likelihood of extinction over time (Harmon and Braude^74^). Populations with a small effective population size have a higher proportion of deaths attributable to the same individual, compared with populations that have a large population size. Thus, the loss of genetic diversity is greater in populations with small effective population sizes. Geographically isolated populations increase rates of inbreeding. Importantly, inbreeding causes inbreeding depression; to avoid this, an *N*_e_ value of > 100 (N < 5 generations) has been suggested^75^. In the present study, the *N*_e_ value was < 100 in the UC and CG groups; thus, efforts to avoid inbreeding depression are needed. In particular, because the UC population exhibits a significant inbreeding coefficient, an active conservation strategy is required. Although the UC population is highly genetically diverse, it inhabits two small regions in the water system; therefore, the long-term effects of inbreeding cannot be ignored. Therefore, population expansion is necessary to increase the genetic diversity of the population and reduce inbreeding.

Frankham et al.^75^ suggested that *N*_e_ > 1000 is needed to maintain evolutionary potential; in all populations of *P. nigra*, *N*_e_ was in the range of 52–301. The species is unlikely to become extinct in the near future but may lose its ability to evolve over time, thus limiting its long-term survival^75^. Therefore, conservation and restoration efforts are suggested for specific populations, as well as all resident populations to maintain their evolutionary potential.

### Genetic structure and gene flow for wild and restoration populations

The nine populations were divided into two water system groups based on genetic structure; DAPC, PCoA, and genetic differentiation indices also supported this population structure (Figs. 2 and 3). An understanding of fish population structure is essential for conservation^76,77^. The MG population was clearly distinct from the Geumgang River water system populations and exhibited genetic differentiation. The populations of the Geumgang River and Mangyeonggang River were geographically separated, which led to genetic differentiation, consistent with the findings in previous studies^45^.

The six populations [OC, ND, YD (2019 and 2012), JJ, and CG] of the Geumgang River water system are connected and exhibit minimal genetic differentiation from each other. Notably, despite its geographical independence, the UC population exhibited minimal genetic difference from the Geumgang River water system populations. This observation was supported by the AMOVA findings of very low genetic variation between the Geumgang River and Ungcheoncheon Stream water system populations.

The UC population has a history of reintroduction after extinction in an area. At the time of restoration, it was reintroduced using fish from the Geumgang River water system population, although this approach has not been confirmed by official records. ABC analysis strongly supported a scenario in which the UC population originated from the Geumgang River population.

Additionally, we observed gene flow among the seven populations, which supported our hypothesis regarding the origin of the UC population (restoration population). The observed gene flow moved from the four Geumgang River water system populations (OC, YD (2019), CG, and ND) to the UC population. Only one Geumgang River water system population, the JJ population, did not show evidence of migrating to the UC population, suggesting that the UC population was derived from the other four populations.

With the exception of the UC population, gene flow existed only within each group; there was almost no gene flow among the UC, MG, and JJ populations. The five populations in the Geumgang River water system showed no evidence of genetic connectivity despite geographical connection. A possible explanation is that gene flow among these populations has been interrupted through the construction of dams and weirs, which presumably caused habitat fragmentation (Fig. 1). An alternative explanation is that there is a one-way connection from upstream to downstream within the Geumgang River water system populations. Generally, the flow of genes within a river is from upstream to downstream, which is the direction of drainage^78^. However, no downstream unidirectional gene flow was observed in this study, although this may be related to sampling bias. In the present study, the JJ, ND, CG, and YD population habitats were tributaries of the main Geumgang River,upstream gene flow was blocked by dams and weirs. Despite this blockage of upstream flow, downstream movement remains possible. BayesAss software only detects migrations of recent generations; thus, the small number of samples may have hindered the detection of obvious gene shifts. Although each population within the Geumgang River water system could move freely before interruption by dams and weirs, the habitat fragmentation caused by their creation may accelerate population extinction^14^. Therefore, considering the results of inbreeding and effective population size among fragmented populations, conservation should be prioritized.

### Conservation implications

*P. nigra* lives in the Geumgang River, Mangyeonggang River, and Ungcheoncheon Stream water system in Korea; it practices brood parasitism in the spawning grounds of *C. herzi*. *P. nigra* cannot reproduce where *C. herzi* does not live^27,79^. Dams and dikes have been constructed in all habitats of this species, and it has already been exterminated once in the Ungcheoncheon Stream water system because of anthropogenic activities. Therefore, a reintroduction strategy was used to restore the Ungcheoncheon Stream water system population, resulting in successful restoration.

Endangered species conservation is intended to increase effective population size by maintaining genetic diversity and improving gene flow^70^. Despite these efforts, the lack of genetic information regarding the UC population has prevented effective conservation planning. Additionally, the genetic structures of the Geumgang River and Mangyeonggang River water system populations were not identified, hindering the selection of populations for restoration and the identification of populations that require conservation. Lack of prior knowledge regarding these genetic structures is a potential threat that could hasten species extinction through delayed conservation management strategies^14,38,67^.

To restore the UC population, fish were artificially propagated using the Geumgang River water system population, which was the most representative population; however, source of the Geumgang River water system population is unknown. Therefore, microsatellite markers were developed to identify *P. nigra* genetic diversity and population structure^46^. In the present study, the genetic structures of the nine populations were determined to provide basic information for use in the selection of priority conservation populations. The UC, OC, and YD populations have high genetic diversity, but the inbreeding issue should be addressed. Additionally, the CG, JJ, and ND populations have a disconnected genetic flow, indicating that conservation efforts require improvement. Because the MG population is a genetically differentiated population, strategies are needed to increase the size of the current effective population and preserve its habitat. Additionally, genetic markers such as single-nucleotide polymorphisms and larger sampling strategies can improve assessments of genetic structure and genetic diversity in newly discovered populations, facilitating the formulation of appropriate conservation plans. Ultimately, the management of inbreeding issues and maintenance of appropriate genetic diversity are essential for the continued conservation of *P. nigra*; our results provide the basic information needed for efficient conservation efforts.

## Supplementary Information

Supplementary Tables.

## Data Availability

All genotypes and related information were available upon request to the authors (e-mail: kimkangrae9586@gmail.com).
