# Supplementary material for: Population structure and genetic diversity of the endangered fish black shinner Pseudopungtungia nigra (Cyprinidae) in Korea: a wild and restoration population
Source: Sci Rep. 2023 Jun 15;13:9692. doi: 10.1038/s41598-023-36569-4 (PMC10272174; doi:10.1038/s41598-023-36569-4)
Supplement: Supplementary file 1 — Supplementary Tables. [file 41598_2023_36569_MOESM1_ESM.docx]

**Table S1**. Sampling sites and numbers of individuals in the study.

| Location | Code | Water system | N | Date |
| --- | --- | --- | --- | --- |
| Okcheon | OC | Geumgang River | 30 | 2019 |
| Mujunamdaecheon Stream | ND | Geumgang River | 30 | 2019 |
| Chogangcheon Stream | CG | Geumgang River | 30 | 2019 |
| Yudeungcheon Stream | YD (2019) | Geumgang River | 30 | 2019 |
| Yudeungcheon Stream | YD (2012) | Geumgang River | 21 | 2012 |
| Jujacheon Stream | JJ | Geumgang River | 30 | 2019 |
| Ungcheoncheon Stream | UC | Ungcheoncheon Stream | 30 | 2019 |
| Mangyeonggang River | MG (2019) | Mangyeonggang River | 30 | 2019 |
| Mangyeonggang River | MG (2008) | Mangyeonggang River | 19 | 2008 |

N: number of samples.

**Table S2**. Combined microsatellite multiplex PCR sets for *P. nigra.*

| Set  No. | Locus | Primer sequence (5′→ 3′) | Motif  repeat | Size range (bp) | Dye label | Reference |
| --- | --- | --- | --- | --- | --- | --- |
| 1 | PN147 | F: GGCTTATGTGGCTTCGGATAC  R: AGGTGAGCCTGAGAGAGAAG | (AC)_9_ | 122-128 | FAM | Bang et al., 2020 |
|  | PN123 | F: GGGACACACTTAGCAAGCCT  R: AGCCAGTGAGATTGAAAGACCA | (GT)_10_ | 213-223 | FAM |  |
|  | PN92 | F: CCGTGCTCATATACAGTCCTC  R: CCGCATTGTTCCTCCGATTG | (CA)_10_ | 258-266 | FAM |  |
|  | PN28 | F: CTCTCCAAATATAGTGCCATCCA  R: ATCCTCCTTTCACCCTCCGT | (AC)_10_ | 237-247 | FAM |  |
|  | PN88 | F: CAACAGGCTCCACGATTGC  R: CTGCCCTCGGAAATAAGATGG | (AC)_7_ | 176-186 | HEX |  |
|  | PN98 | F: CAGGATGAGTCCATCGTCTC  R: GCTCAGAAGTGACCGACAGA | (GT)_10_ | 281-301 | HEX |  |
|  | PN23 | F: GGCACTCAAGGATAATCTGAAC  R: TGTATCCGGCCTCTGTGTAG | (GT)_10_ | 200-212 | TAMRA |  |

**Table S2**. (Continued)

| Set  No. | Locus | Primer sequence (5′→ 3′) | Motif  repeat | Size range (bp) | Dye label | Reference |
| --- | --- | --- | --- | --- | --- | --- |
| 2 | PN4 | F: TCGGTGGATGCGAGGAATAC  R: TTCCGCCTGTCAGTCAAGAC | (AC)_6_ | 190-202 | FAM | Bang et al., 2020 |
|  | PN125 | F: CTGCACTCACTTCCATAGACG  R: AACAGTGACTCAGTGGACAGG | (TG)_9_ | 166-184 | FAM |  |
|  | PN124 | F: GAGACGCACGACTGATGAAG  R: GGCTAACAGGGCGATTGATTG | (AC)_10_ | 246-258 | FAM |  |
|  | PN75 | F: CCTGCATCCATGCCGTATAG  R: GCTGTTATAGCGCTGATGATAG | (AC)_9_ | 199-217 | HEX |  |
|  | PN126 | F: CCACACTACTGAGACTAAACTG  R: TGACAGACCATCTTGCATTCTG | (GT)_9_ | 176-186 | HEX |  |
|  | PNms197 | F: CGTCACGCACTAATCAGGAACTG  R: ACTGCATCAACCCTCAGCGA | (GT)_16_GCAC(GT)_9_ | 137-195 | TAMRA | Kim, 2012 |

**Table S2**. (Continued)

| Set  No. | Locus | Primer sequence (5′→ 3′) | Motif  repeat | Size range (bp) | Dye label | Reference |
| --- | --- | --- | --- | --- | --- | --- |
| 3 | PN94 | F: CAGCACTTCCTGTTGGAGGT  R: GGAGTATGTAAGGGGTCCAGA | (CT)_8_ | 98-114 | FAM | Bang et al., 2020 |
|  | PN154 | F: CCCACCAGATTGTATCGGGAT  R: TCTGTGTTAACGGTGCAGGAA | (TC)_10_ | 161-169 | FAM |  |
|  | PN71 | F: TCCACACCGCACCACAATG  R: CGCATCACACACACCGTAAC | (TG)_9_ | 214-226 | FAM |  |
|  | PN70 | F: GTCGCCAACAGCTCATCTTG  R: GCTGAGAGCTTGAGTCATCAC | (GT)_9_ | 268-278 | FAM |  |
|  | PNms246 | F: TATGTGCAGGGAAGACGGCA  R: TGAGCACTCACACCCACCAA | (GT)_14_ | 194-216 | HEX | Kim, 2012 |
|  | PN64 | F: TCAACACAACGGACAGATTCG  R: CTATAAGTACTGCCCCGTGCT | (AC)_8_ | 249-255 | HEX | Bang et al., 2020 |

**Table S2**. (Continued)

| Set  No. | Locus | Primer sequence (5′ → 3′) | Motif  repeat | Size range (bp) | Dye label | Reference |
| --- | --- | --- | --- | --- | --- | --- |
| 4 | PNms152 | F: AAGGCTGAACTGAGCCCAGAA  R: GCGGAGCAGAAATCATCGTG | (CA)_10_ | 158-198 | FAM | Kim, 2012 |
|  | PN139 | F: GTGCGAGTGAACGACTGATG  R: ACTTGTGCCTGCTGCTAGAG | (TG)_8_ | 208-218 | FAM | Bang et al., 2020 |
|  | PN133 | F: AGACAGCTCAGGACCAATCC  R: CCACTAACTGACGTGCAGAAT | (AC)_9_ | 226-234 | FAM |  |
|  | PN99 | F: AGAGGAGGTCAGATGCTATGC  R: GCCAATAATCCGTGCCGATAG | (TC)_8_ | 221-245 | HEX |  |
|  | PNms560 | F: ACATCAGGCCTGTCATGGTGA  R: GCGTACATGGAAACACGACG | (CA)_37_ | 123-179 | HEX | Kim, 2012 |
|  | PNms180 | F: CACGGACTCGGCTGTTTTCA  R: GGGATGTTTACTTTTAAAGGGCTCA | (GT)_12_ | 193-227 | TAMRA |  |
